# Immune cell labelling and tracking: implications for adoptive cell transfer therapies

**DOI:** 10.1186/s41181-020-00116-7

**Published:** 2021-02-03

**Authors:** Filippo Galli, Michela Varani, Chiara Lauri, Guido Gentiloni Silveri, Livia Onofrio, Alberto Signore

**Affiliations:** 1 Nuclear Medicine Unit, Department of Medical-Surgical Sciences and of Translational Medicine, Faculty of Medicine and Psychology, “Sapienza” University of Rome, Rome, Italy; 2 Medical Oncology B, Department of Radiology and Pathology, “Sapienza” University of Rome, Rome, Italy

**Keywords:** Cell labelling, Molecular imaging, Immunotherapy, Adoptive cell transfer

## Abstract

**Background:**

The understanding of the role of different immune cell subsets that infiltrate tumors can help researchers in developing new targeted immunotherapies to reactivate or reprogram them against cancer. In addition to conventional drugs, new cell-based therapies, like adoptive cell transfer, proved to be successful in humans. Indeed, after the approval of anti-CD19 CAR-T cell therapy, researchers are trying to extend this approach to other cancer or cell types.

**Main body:**

This review focuses on the different approaches to non-invasively monitor the biodistribution, trafficking and fate of immune therapeutic cells, evaluating their efficacy at preclinical and clinical stages. PubMed and Scopus databases were searched for published articles on the imaging of cell tracking in humans and preclinical models.

**Conclusion:**

Labelling specific immune cell subtypes with specific radiopharmaceuticals, contrast agents or optical probes can elucidate new biological mechanisms or predict therapeutic outcome of adoptive cell transfer therapies. To date, no technique is considered the gold standard to image immune cells in adoptive cell transfer therapies.

## Introduction

Recent immunotherapeutic approaches demonstrated that it is possible to boost our immune system against cancer, either by re-programming tumor infiltrating immune cells or by injecting ex vivo expanded immune cell subpopulation like in adoptive cell transfer. Being able to track these immune cells in vivo is of great importance in elucidating the biology of tumor microenvironment and in evaluating the efficacy of cell-based therapies (Bissell and Radisky 2001; Fridman et al. 2012). This is particularly important in patients eligible for immunotherapies where conventional criteria and imaging modalities ([^18^F]FDG-PET/CT) are not able to differentiate true progression from pseudoprogression. Indeed, an increase of tumor infiltrating immune cells cause both an increase in tumor size and an increase of glucose uptake when imaging with [^18^F]FDG (Vrankar and Unk 2018). Also, in pre-clinical research, differential diagnosis between true progression and pseudoprogression represents a major issue, and assessment of therapy efficacy of new drugs becomes very challenging. This is usually performed by indirect measurement of some biomarkers, immunohistochemistry on excised organs or tissue biopsies or by other techniques depending on the pre-clinical/clinical setting and disease (Borcoman et al. 2018).

Molecular imaging is a non-invasive approach that allows us to follow the fate of a target cell in vivo, in real time and, in some cases, it might also be used to predict therapy response.

Indeed, cells can be directly or indirectly labelled with radiopharmaceuticals, contrast agents or fluorescent probes for nuclear medicine, magnetic resonance or optical imaging (Kircher et al. 2011; Zeelen et al. 2018).

Direct labelling consists in the ex-vivo labelling of cells with an agent that remains trapped inside the cytoplasm, then labelled cells are injected in the subject. There is no need to genetically modify cells and, for this reason, this approach is commonly used in clinical practice (as for example radiolabelled white blood cells for imaging infection/inflammation) (Signore et al. 2018). However, the labelling agent is diluted with time as cells divide and proliferate resulting in a loss of sensitivity. In some cases, like when ^111^In-oxiquinolon (^111^In-oxine) is used, cells may die due to radiolysis or the agent can be lost, increasing the signal from the circulation and decreasing the target-to-background ratio. These issues do not allow us to perform imaging at very late time points or to evaluate cell proliferation, activation or death.

By indirect labelling we refer to in vivo targeting of specific antigens expressed by immune subtypes or to the use of reporter genes. The former approach is the simplest as we only need a labelled monoclonal antibody (mAb) or peptide that is able to bind to target cells in vivo, without the need of ex-vivo or genetic manipulation. Limitations of this approach are represented by expression of the target antigen by other specific subpopulations or physiologic uptake of the label by major organs. These limitations can be overcome by the use of reporter genes like bacterial thymidine kinases that are stably introduced in cells of interest under the control of a given promotor. Upon different stimuli, these genes are transduced in specific receptors, proteins or enzymes that are led to the internalization or activation of a labelled agent. This allows us to perform imaging at very late time points with virtually no time limit, as the injection of the agent can be repeated as many times we need. We can then follow the fate of specific cell subtypes or their progeny until they die, as the gene can be passed with cell division, and evaluate cell expansion or other biological processes. Unfortunately, this approach is difficult to translate in humans due to safety and regulatory issues as there is the need of genetic manipulation of cells and unique agents or targets depending on involved immune subpopulations in different diseases.

In the present review, we will report the state of the art of cell labelling techniques with potential applications in chimeric antigen receptor (CAR) cell therapies.

## Chimeric-antigen-receptor (CAR) cells

### CAR-T cells

CAR-T adoptive cell therapy is currently approved by the Food and Drug Administration (FDA) in various haematological malignancies, including certain types of lymphomas and in acute lymphoblastic leukaemia. It may also represent a promising immunotherapy against solid tumors (Sur et al. 2020). CARs are engineered fusion proteins that recognize carbohydrate and glycolipid antigens bypassing major-histocompatibility complex (MHC) restrictions. There are presently four CARs generations, which share the same extracellular domain, hinge/spacer and transmembrane domain, but present distinct intracellular signalling motifs. First generation CARs present a CD3ζ signalling domain characterized by the immunoreceptor tyrosine-based activation motifs (ITAMs) from the CD3ζ chain only. In order to improve upregulation of anti-apoptotic gene expression, cytokine secretion (in particular, IL-2), T-cell expansion and persistence (Chen and Yang 2017; Sadelain et al. 2003), second generation CARs including members of the immunoglobulin superfamily (IgSF), such as CD28 and CD287 or Inducible T-cell COStimulator (ICOS), members of the tumor-necrosis factor receptor super family (TNFRSF), such as 4-1BB (CD137), OX40 (CD134), CD27 and DAP10. Third generation CAR T cells present co-stimulatory signals, including CD28 and 4-1BB. Fourth generation CARs express various co-stimulatory components and are employed in universal cytokine killing T cells (TRUCKs), which mediate innate immune response against tumors. Innate responses exert their anti-cancer activity via proinflammatory cytokines, such as IL-12, as well as antigen-independent elimination of hostile microenvironmental cells expressing IFN-γR. CAR-T cells are engineered T-lymphocytes which are infused in patients to obtain an anti-tumor immune response. Manufacturing of CAR-T cells is accomplished via a multistep process, which includes collection, activation, genetic modification and expansion of T cells. In detail, blood mononuclear cells (PBMCs), enriched for various membrane receptors, such as CD4, CD8, CD25 or CD62L, are first obtained from peripheral blood via leukapheresis. T-cell activation is obtained after 3–21 days of ex vivo culture using anti-CD3 antibody in combination with either anti-CD28 antibody co-stimulation or via co-culture with PBMCs (Maher et al. 2002; Carpenito et al. 2009). CAR-T cells are expanded by stimulation with IL-2, IL-7 and/or IL-15 and are “armored” with targeting antigens, such as pan–B-cell CD19 antigen expressed on tumor cells and on antigen-presenting B cells (APCs). Production of CAR-T cells involves the ex vivo enrichment and growth of T lymphocytes that are genetically engineered under GMP conditions. Genetic engineering is achieved via the use of viral (lentivirus, γ-retrovirus, adenovirus or adeno-associated virus) and non-viral vectors, such mRNA delivered via electroporation, synthetic DNA or mRNA transposon systems (Wang and Rivière 2016; Izsvák et al. 2010). The introduction of suicide genes transfecting T cells with a late-stage apoptosis pathway molecule iCasp9/iFas constructs via plasmids is useful for improving the safety of the CAR-T cell therapy (Eyquem et al. 2017). However, limited T cell persistence, antigen loss, immunosuppression, T cell exhaustion and activation-induced cell death (AICD) remains the most relevant hurdles of CAR-T cell treatment. Recently, to overcome some of these limitations, an innovative technology platform called UniCAR has been explored. Recombinant targeting modules (TMs) are engineered with a single epitope (UniCAR epitope), which recognize the tumor site on one side, and UniCAR-T cells on the other. Therefore, after infusion with the TMs, UniCAR-T cells are cross-linked with tumor cells via TMs. By stopping the TMs infusion, the UniCAR-TM complex disassembles and the activity of the UniCAR-T cells is blocked. In this way, the activity of UniCAR T cells can be controlled by the infusion of TMs and stopped in case of side effects or after successful therapy (Bachmann 2019).

### CAR-NK cells

**Fig. 1:**
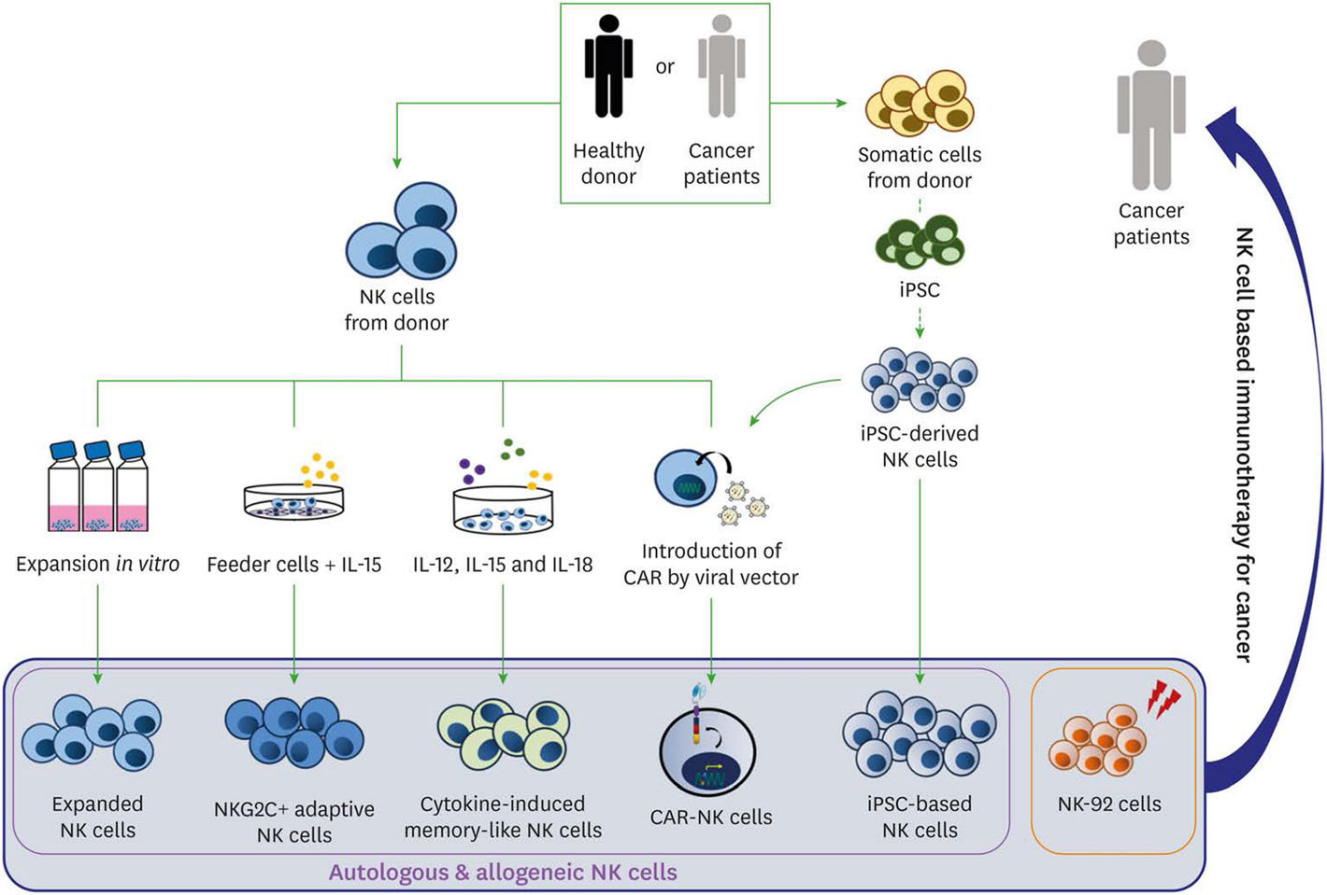
Methods of NK cell-based immunotherapy for cancer. Types of NK cells used for cancer immunotherapy. Allogeneic NK cells and autologous NK cells, manipulated with cytokines (IL-12, IL-15, and IL-18) and CAR, resulted in various types of NK cells for cancer immunotherapy. Somatic cells from the donor could be used as functional iPSC-based NK cells. Irradiated NK-92 cell line also served as an important resource for NK cell-based cancer therapy (Shin et al. 2020)

Natural killer (NK) cells have innate antitumor properties via activating or inhibitory receptors that recognize self-human leukocyte antigens (HLAs) expressed on all the cell membranes (Sanseviero 2019). The absence or HLA or the present of aberrant ones, induce the NK killing. CAR-NK cells adoptive therapy is classified as advanced therapy medicinal product (ATMP) in Europe. Absence of graft versus host disease (GVHD) adverse effect is one of the most important advantages of the CAR-NK therapy (Glienke et al. 2015). On the other hand, the lack of rearranged specific receptors on NK membrane actually represents a disadvantage. Their receptors are activating if they recognize a positive signal such as abnormal antigens or missing-self, instead are inhibitory if they recognize self-MHC antigens on cells surface. NK cells may be genetically customized in order to improve the persistence in circulation and the cytokines release necessary for their autocrine proliferation and survival (Imamura et al. 2014; Liu et al. 2018). Again, the insertion of suicide gene into CAR-modified effectors represents a strategy to improve the safety of NK cell immunotherapy and to delay the recognition of some antigens expressed both in cancer cells and healthy cells. Several pre-clinical studies conducted on CAR-NK cells have been demonstrated their relevant role against B and T malignancies, multiple myeloma (Ruggeri et al. 2002), glioblastoma, neuroblastoma, epithelial cancers and melanoma (Leivas et al. 2016; Mehta and Rezvani 2018). NK cells directed against CD19 and CD20 were studied in B malignancies, glioblastoma and other cancers (Rezvani et al. 2017; Shimasaki et al. 2012; Sahm et al. 2012; Zhang et al. 2015). Despite their potential, there are no CAR-NK based therapies approved for human use yet, but strategies for their application are the same as for CAR-T cells (Fig. 1) (Shin et al. 2020). For this reason, molecular imaging approaches might help in understanding their in vivo biodistribution and efficacy in examined cancer types.

## Imaging cell trafficking

Morphological and functional evaluation of organs and tissues can be performed by imaging approaches that offer the advantage of providing selective analysis of tumors clonal component, labelling specific antibodies both in soluble form and linked to T lymphocytes. The topographic tissues distribution of CAR cells for solid tumors, the dosimetry and micro-dosimetry for all the single sections provide clinical-instrumental helpful details and allow the evaluation the benefits from CAR cells therapy. The reference to a positivity score for each individual lesion allows physicians to estimate an accurate correlation between the tracer uptake and the response to CAR cell treatment and provides to identify the possible cut-off value above which to expect the greatest probability of response. Micro-dosimetry and post-acquisition reconstruction of each single lesion can provide a map of the antigen-specific tumor clone bio-distribution. In case of low positive or negative neoplastic lesions CAR cell therapy has to be avoided and replaced by other approaches (i.e. radiotherapy and/or chemotherapy). The binding kinetics between the antibody (Ab) linked to the CAR and the corresponding antigen (Ag) is different from the soluble Ab-Ag binding kinetics characterized by dissociation costant Kd = [AbAg]/[Ab] [Ag]. Moreover, direct and indirect cellular interaction mechanisms, which guarantee antineoplastic activity, interfere with binding kinetics. In this scenario, researchers have been investigating several molecular imaging approaches to gather more information about CAR cell trafficking and establish non-invasive diagnostic techniques for therapy-decision making, efficacy evaluation and follow-up.

## Nuclear medicine imaging

**Fig. 2:**
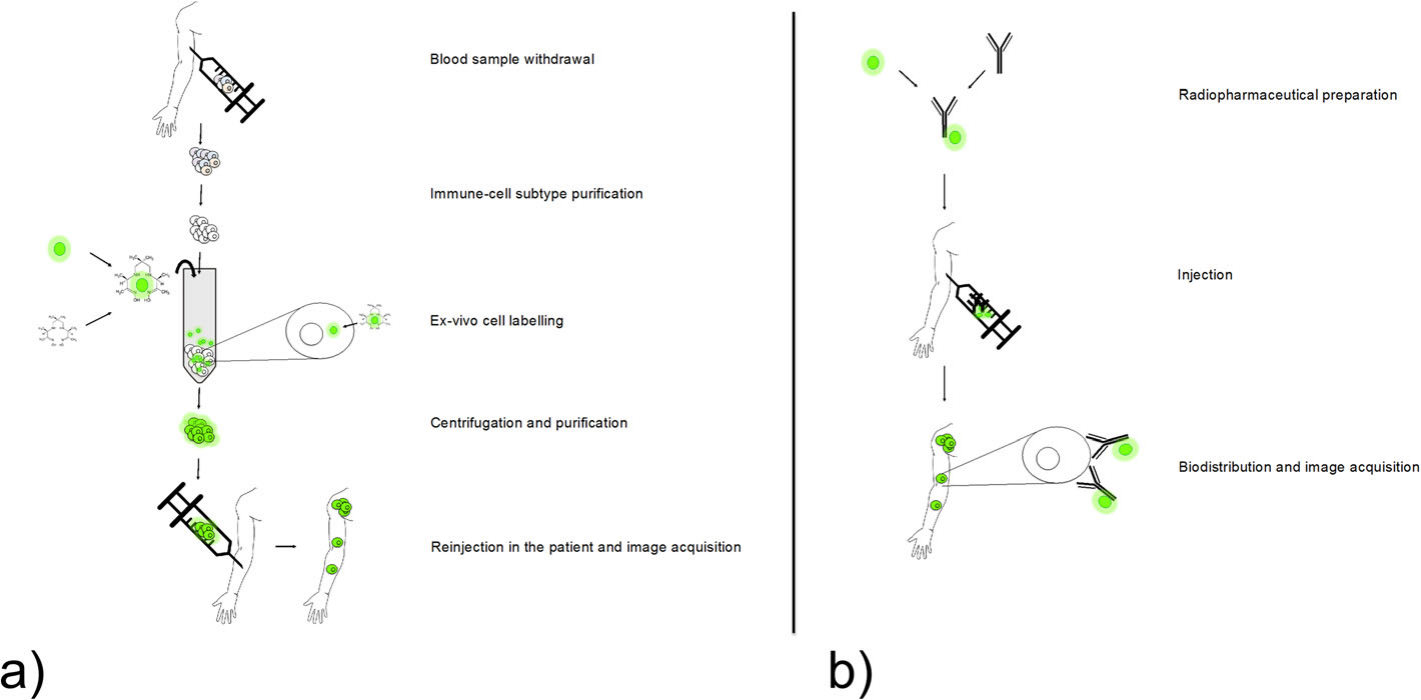
a) Direct (ex-vivo) and b) indirect (in vivo) radiolabelling procedure of an immune cell subtype in humans for nuclear medicine applications

Direct and indirect cell labelling and tracking with radiopharmaceuticals is a well-established methodology (Fig. 2).

Indeed, ^111^In-oxine has been used in clinical practice for more than 30 years to radiolabel white blood cells (WBC) for imaging of infection and inflammation (Roca et al. 2010; Teiler et al. 2020). Oxine is a lipophilic molecule that chelates indium-111 and is able to freely diffuse inside the cell through the plasma membrane. Once inside, the radioisotope binds to intracellular proteins and remains trapped in the cytoplasm. Also, exametazime (HMPAO) can be used for the same purpose, but it chelates technetium-99 m and shows a slightly different biodistribution due to biliary excretion with consequent detection of bowel activity (de Vries et al. 2010). A PET alternative is [^89^Zr]Zr-oxine or pyruvaldehyde-bis(N4-methylthiosemicarbazone) (PTSM) that chelate copper-64 or even ^64^Cu-gold nanoparticles (Weist et al. 2018; Adonai et al. 2002; Li et al. 2014). When WBCs scintigraphy is performed, there is no distinction of the different immune subpopulations that are injected in the patients and the major contribution comes from granulocytes that infiltrate infected foci. As new specific immune or cell-based therapies are developed, there is the need to specifically image a precise immune cell subtype. Indeed, it might be even necessary to distinguish between T cell subsets (i.e. CD4+ vs CD8+) and their radiolabelling with these radiopharmaceuticals has already been investigated. This can be achieved by prior purification of the desired subset from peripheral blood mononuclear cells (PBMCs), followed by radiolabelling. In mice, [^111^In]In-oxine labelled T cells were able to migrate into tumor lesions without evident toxicity (Cussó et al. 2014). Same findings were observed in humans with radiolabelled NK cells injected in a patient affected by metastatic renal carcinoma that had to undergo adoptive NK cell transfer therapy. They were able to image 50% of known lesions by SPECT/CT, however, they also reported a high circulating activity due to released indium-111 (Meller et al. 2004). It appears that despite overall cell viability is not substantially affected, altered biodistribution and radiotoxicity have been observed, representing a major limitation of this approach. This is particularly relevant for ^111^In-oxine labelled cells as indium-111 induces radiolysis leading to low target to background ratios and image misinterpretation even if renal excretion of indium-111 mitigates this effect. It must be considered that the problem of altered biodistribution can arise with all the labeling techniques mentioned in this review, especially when ex-vivo expansion is performed. This is because the use of any labelling agent or cell manipulation could influence the expression of some surface markers that modify the trafficking of the cell line studied. To avoid this limitation, specific in vitro assays and preclinical in vivo studies should be conducted before human studies. Also, macrophages were radiolabelled with [^111^In]In-oxine after ex vivo differentiation, but after injection in a metastatic patient, only 1 lesion in a cohort of 15 patients was imaged by SPECT (Quillien et al. 2001). The interplay between different immune subsets could also be studied by dual isotope imaging. Cells can be labelled with different isotopes and SPECT images can be acquired and corrected with proper algorithms. Although this might be feasible in small animals, it will be difficult to implement in clinical practice with current technologies.

The low tumour/background (T/B) ratio observed with [^111^In]In-oxine labelling can be overcome using isotopes for PET imaging like fluorine-18, carbon-11, zirconium-89 or copper-64 (Weist et al. 2018; Adonai et al. 2002; Quillien et al. 2001). Carbon-11 and fluorine-18 are less suitable due to their short half-life, since it has been observed that [^18^F]FDG labelled NK cells remains trapped in the lungs at early time-points. [^64^Cu]Cu-PTSM and ^89^Zr-oxine have more suitable half-life, however, studies in mice confirmed a significant efflux of the radioisotope from the cytoplasm. The free zirconium-89 accumulates in the bone marrow with consequent increase of radiation burden and image misinterpretation. Li et al., tried to solve this issue by labelling T lymphocytes with ^64^Cu-gold nanoparticles through electroporation but their study had no real follow up other than in pre-clinical tumor models (Melder et al. 1993). Albert et al. developed a nanobody (nb)-based TM directed against EGFR overexpressed on tumor cells. The TM was radiolabelled with ^64^Cu and ^68^Ga to analyse the biodistribution, the targeting and the stability of the TM and of the UniCAR-TM complex. Results showed the efficient tumor targeting both in vitro and in vivo of UniCAR T cells complexed with TM and the tumor cell lysis. They demonstrated as the killing of tumor cells correlates with the TM concentration and as the fast elimination of TM from the body provokes a shutdown of UniCAR T cells (Albert et al. 2017).

Indirect labelling approaches do not suffer from loss of the labelling agent from the cells and the dilution effect does not take place as the transfected gene is duplicated upon cell division. This way T/B ratio is greatly increased and it is also possible to monitor cell proliferation rate as the signal increases with the number of new cells. Usually the inserted gene encodes for specific enzymes or receptors that facilitate the transport of the labelling agent inside the cells where it remains trapped. Unfortunately, this strategy requires ex vivo genetic manipulation and the development of specific reporters and agents depending on the desired application. For this reason, specialized equipment and training are required and this approach might suffer of regulatory issues when translating it in humans. Sterility and safety of ex-vivo manipulated cells are always a crucial issue for radiopharmacies that often do not have the facilities for cell manipulation. In direct labelling approaches this has been solved by the introduction of specific kits that allows technician and radiopharmacists to radiolabel cells without the need of dedicated equipment, but for indirect approaches this has not been achieved yet (Auletta et al. 2019). Lentiviral vectors are usually used to transfect the gene that integrates in the genome of target cells under the control of specific promotor that is activated by proliferation stimuli. The use of viruses is another possible source of risks as the gene may integrate in non-target sites with mutagenic potential (insertional oncogenesis) (Schlimgen et al. 2016).

It is also possible to use physical or chemical methods like electroporation, cationic or chemical transfection agents (Galli et al. 2014). The Herpes simplex virus thymidine kinase type-1 (HSV1-tk) has been widely used as a reporter gene for nuclear medicine applications (Park et al. 2012). Its product is a thymidine kinases not present in eukaryotic cells and it is able to phosphorylate specific nucleosides that can be injected in a radiolabelled form like 9-[4-[^18^F]3-(hydroxymethyl)butyl]guanine ([^18^F]FHBG), 2-deoxy-2-[^18^F]5-ethyl-1-D-arabinofuranosyluracil ([^18^F]FEAU) or 2-deoxy-2-[^18^F]5-iodo-1-D-arabino-furanosyluracil ([^18^F]FIAU) (Gambhir et al. 1998). After phosphorylation these compounds are not able to exit the cell anymore and allows the detection of the specific subset transfected with the gene. Since they are rapidly excreted, signal from major organs is greatly reduced, thus increasing the T/B ratio. Unfortunately, those analogues are not able to pass the brain barrier, thus preventing to image brain metastasis. Epstein-Barr virus (EBV)-specific T cells transduced with HSV-tk reporter gene were used to follow their infiltration of EBV-positive tumors in SCID mice (Koehne et al. 2003). After adoptive cell transfer, mice received an intravenous injection of [^131^I]FIAU or [^124^I]FIAU to perform SPECT or PET imaging respectively. The injection can be repeated as many times as needed to monitor the number of T cells in tumor lesions over time. Authors reported that their number closely correlated with the radioactive signal from the lesions. Moreover, a higher degree of infiltration corresponded to a slower tumor progression. This is a promising and potentially feasible approach that could be translated in humans after solving all issues related to safety and regulations. One advantage of the use of HSV1-tk is that death of transfected cells can be induced by ganciclovir administration. Upon phosphorylation by HSV1-tk it become toxic causing cell death (suicide gene). The sodium-iodine symporter (NIS) is an alternative to HSV1-tk and imaging can be performed after administration of radioactive iodine, technetium-99 m or [^18^F]tetrafluoroborate ([^18^F]BF4) (Jauregui-Osoro et al. 2010). However, this symporter is physiologically expressed in other organs like the thyroid, salivary glands and stomach, therefore, it is possible to observe unspecific uptake of the radiopharmaceutical at these sites. Preliminary studies with this reporter gene have been performed in lymphocytes and other cell types in murine models and demonstrated the feasibility of this approach. In humans, the first study of indirect labelling with a gene reporter was performed in 2009 in a patient affected by glioblastoma (Yaghoubi et al. 2009). The patient was selected to undergo adoptive cell transfer therapy with ex vivo expanded autologous cytolytic CD8+ T cells, genetically engineered to express the interleukin-13 zetakine gene (therapeutic gene, encoding a receptor protein that targets the T cells to the tumor cells), and the HSV1-tk reporter gene. [^18^F]FHBG PET imaging demonstrated the feasibility of this approach, as authors were able to image not only the already known lesion, but also a secondary lesion. This also demonstrated the infiltration of metastases by T cells.

Whether these methods could be used also to predict outcome of immune or cell-based therapies is still a question mark, as it would require more than one injection of genetically manipulated cells and it is not known if the degree of infiltration prior to therapy correlates with the response.

**Fig. 3:**
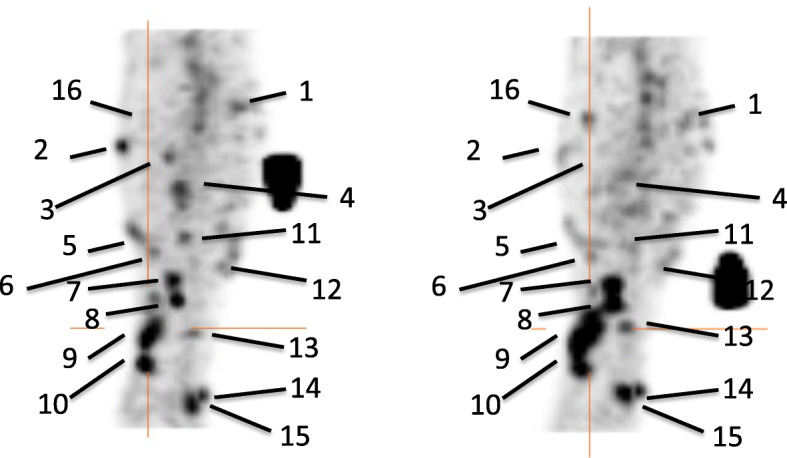
Pre-therapy (left) and post-therapy (right) images of [^99m^Tc]Tc -HYNIC-IL2 uptake in metastatic lesions of a patient. A total of 16 lesions were identified in the affected leg for quantitative analysis. It can be seen that some lesion increased uptake over time, other decreased (Markovic et al. 2018)

Other indirect approaches rely on the use of specific radiolabelled mAbs or peptides that bind to target antigens expressed on the plasma membrane of different cell subsets. This is the simplest way to image trafficking of immune cells in tumors, as there is no need of ex vivo or genetic manipulation. Therefore, from a regulatory point of view this is the most feasible approach. On the other hand, it is not always possible to image only a specific subset as the target antigen might be expressed also by other cell types and the T/B ratio is usually lower in comparison to those obtained with direct or gene reporter strategies. In particular, mAbs have a long circulating half-life and, if not humanized, human anti-mouse antibodies (HAMA) production may occur. This limitation can be overcome also with the use of fragments (Fab/Fab2 or diabodies) that will have a lower molecular weight and more likely different excretion routes. In the literature, many radiopharmaceuticals to image immune cells has been reported and tested also in humans. Among them we may find radiolabelled anti-TNFalpha, anti-CD20, anti-granulocytes mAbs that were mainly used to image cells in inflammatory diseases (Signore et al. 2019). A radiopharmaceutical that has been investigated in patients affected by metastatic melanoma and before and after immunotherapy with ipilimumab is [^99m^Tc]Tc-interleukin-2 ([^99m^Tc]Tc-IL2). This molecule binds to its receptor expressed on activated T cells that are the main effectors of the therapeutic response in patients undergoing immunotherapy (Markovic et al. 2018). A pilot study in 5 patients showed that this radiopharmaceutical is able to detect tumor infiltrating T cells and that lesions with an increased size but decreased SUV were the ones that responded less to therapy (Fig. 3).

Tumor infiltrating NK cell trafficking in undifferentiated tumors has been performed in nude mice with a radiolabelled anti-CD56 mAb since the CD56 is a marker specifically expressed by NK cells (Galli et al. 2015). Imaging of this immune subtype is gaining more importance as new CAR-NK cell therapies are under investigation as an alternative to CAR-T cells with lower toxicity.

Mice bearing a tumor in the right thigh received an injection of human NK cells followed by the injection of the radiopharmaceutical. Imaging was performed up to 24 h demonstrating uptake of the labelled anti-CD56 in tumors that correlated with immunohistochemical staining for NK cells. Moreover, more infiltrated tumors showed more necrotic areas, thus confirming the anti-tumor potential of these lymphocytes.

## Magnetic resonance imaging

**Fig. 4:**
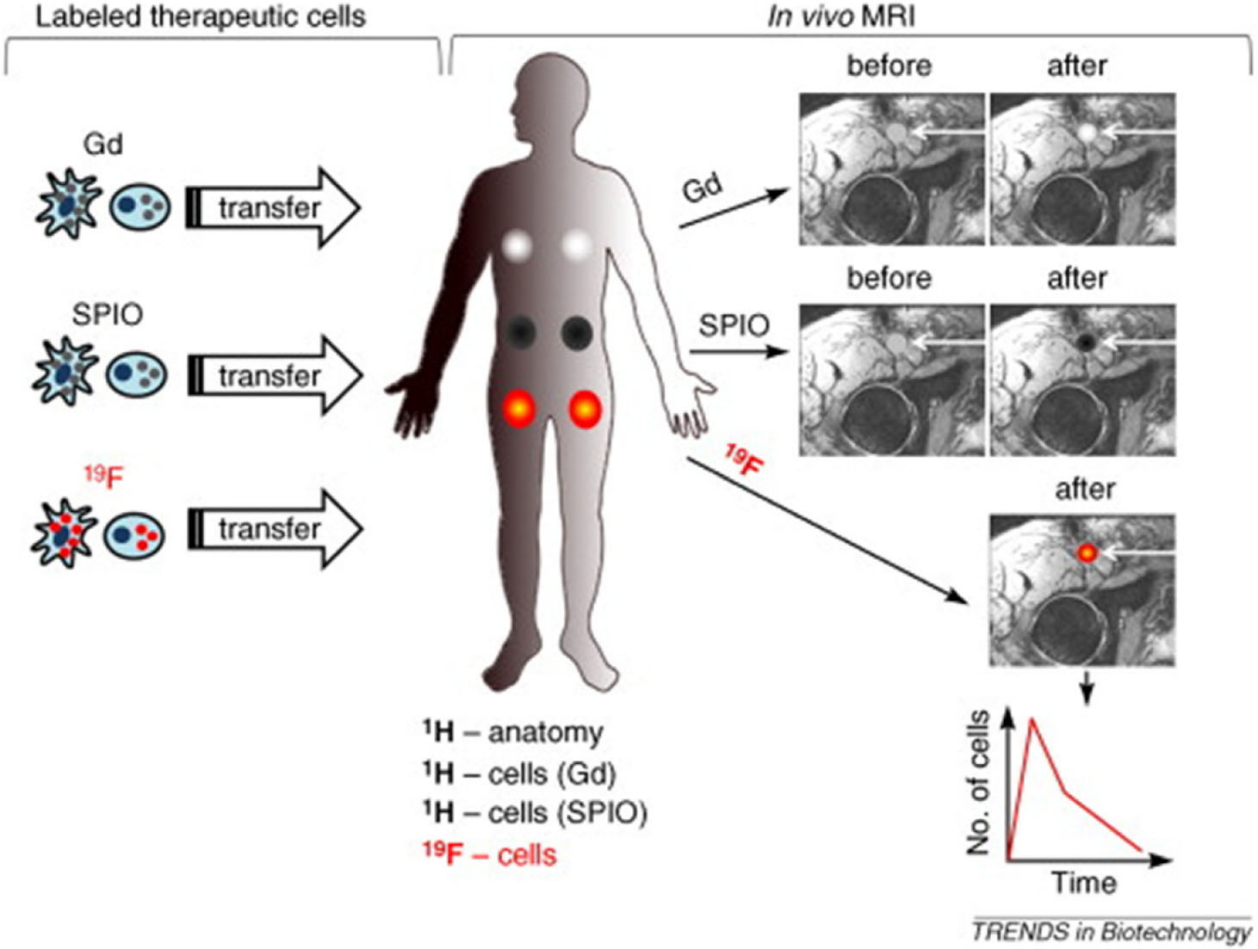
Cell tracking using MRI with contrast agents and ^19^F labels. Therapeutic cells (e.g. dendritic cells or stem cells) are labeled using contrast agents (Gd or SPIO), or a ^19^F label. Conversely, typical anatomical MRI utilizes the ^1^H from H_2_O in tissues. Labeled cells are transferred to the patient by localized transfers, including intradermal or intranodal injections; the patient is then imaged to determine cell localization. Gd and SPIO (superparamagnetic iron oxide) labels typically require “before” and “after” images for localization (white arrows), resulting in a final hyperintense or hypointense spot signal, respectively. With a ^19^F label, imaging can be carried out in a longitudinal manner for ^19^F (specifically the labeled cells) and ^1^H (anatomy) without a “before” image. Furthermore, the total ^19^F signal can be calculated from the in vivo data, providing tracking information for both the localization and number of cells. Such quantitative, unambiguous cell tracking is not possible using standard imaging techniques in conjunction with metal-based contrast agents (Srinivas et al. 2010)

Magnetic resonance imaging has been proposed as an alternative approach to track cells in vivo with the use of contrast agents and without the use of ionizing radiations. The approach is very similar to direct labelling with radiopharmaceuticals as target cells are purified from a patient and ex vivo labelled with a suitable contrast agent. Usually they are paramagnetic contrast agents as molecules that chelates gadolinium(III) and lead to positive contrast in T1-weighted sequences (Rudelius et al. 2003). Unfortunately, concerns about possible toxicity of gadolinium(III) made it a less appealing contrast agent. Alternative compounds are paramagnetic chemical exchange saturation transfer (PARACEST) that works in a manner to enhance image contrast at certain radiofrequencies (Aime et al. 2005). Despite promising results, these agents have been reported not to be sensitive enough to image small number of cells. Therefore, given their higher effect on relaxivity, superparamagnetic iron oxide particles have been replacing conventional contrast agents. They are composed of an iron-oxide core with a polymeric shell. Also, dextran citrate or siloxan can be used (Kim et al. 2011; Moore et al. 1997). The higher sensitivity is caused by the presence of thousands of atoms of iron that lead to enhanced negative contrast in T2-weightd sequences. The size of the particles can be tuned as needed and may vary from 10 nm (ultra-small, USPIO) to more than 1 μm. First reports in humans demonstrated that autologous dendritic cells could be labelled with SPIO and visualized by MRI. In this first report, cells were injected in the lymph node of a patient and were also radiolabelled with [^111^In]In-oxine to confirm their accumulation by SPECT imaging. MRI showed a similar sensitivity to nuclear medicine imaging and this study was followed up by many others also in different immune cell subtypes (Zelivyanskaya et al. 2003). However, different cell subsets show different labelling yield with SPIO. For example, phagocytic cells show greatest incorporation rate, whereas lymphocytes the lowest. Optimization of the labelling methods is being pursued for human application with these immune subtypes and led to the development of methodologies to label even NK cells. This has been achieved by using ferumoxytol, FDA approved USPIO, with the presence of protamine sulphate (PS) or PS and heparin (H) mixtures (Li et al. 2015). Other attempts have been made using mAbs conjugated with SPIO that are eventually internalized in the cells bringing the particles inside with them. This approach has been used with an anti-transferrin-receptor antibody OX-26 conjugated with mono-crystalline iron oxide-46 L or the streptavidin/biotin system to label and track oligodendrocyte progenitor cells after transplantation (Smirnov et al. 2008). Like for radiolabelled mAbs, the development of HAMAs is a well-known clinical issue that limits their use in humans. The need of a humanized version greatly increases the costs of this methodology. Nowadays, there are commercially available agents that can be used to transfect SPIO like Feraheme®, Feridex I.V.®, SuperFect® (Jha et al. 2010). These methods, together with the use of electroporation, can avoid the use of chemical modifications and were used to track tumor infiltrating NK cells in mice bearing HER2/neu positive mammary tumors and other preclinical studies (Daldrup-Link et al. 2005). Also, fluorine-19 perfluorocarbon, despite its low sensitivity, has been proposed as a promising agent to label cells for MRI application, given its low toxicity and negligible background (Janjic et al. 2008). MRI labelling approaches summarized in Fig. 4 have the advantage of being safer, as there are no ionizing radiations (Srinivas et al. 2010). However, the low sensitivity is still a major limitation and from this point of view nuclear medicine imaging is superior and already tested in humans and clinical practice.

## Optical imaging

Optical imaging (OI) is a relatively cheap and easier approach if compare with MRI or nuclear medicine imaging. There is no ionizing radiation and it does not require highly specialized facilities. Indeed, it is very suitable for preclinical research, but its applicability in humans is limited by the low penetration depth of light. The OI methods for in vivo cell tracking included fluorescence imaging (FLI), bioluminescence (BLI), microscopy imaging (MI), like confocal and two-photon microscopy, and diffuse optical tomography (Sutton et al. 2008). The use of intravital microscopy helped in elucidating many biological mechanisms offering high cellular resolution, but it is a very invasive technique limited to a small area and only in accessible tissues, thus not being the most feasible strategy to repeatedly track immune cells in vivo (Weigert et al. 2010)*.* Conventional optical imaging has many similarities with nuclear medicine imaging as the radionuclide is replaced with an optical contrast agent or fluorochrome. This agent should be biocompatible, photostable, PH insensible, with tolerable cytotoxic levels and with low background signal. Therefore, in theory, any mAb or peptide labelled with a fluorescent agent can be used. In the literature, many different probes, like mAbs, peptide, aptamers, nanocrystals or quantum dots, have been proposed to label cells and track them in vivo (Seth et al. 2017). The labelling between these probes and optical contrast agents can be direct/exogenous or indirect/endogenous. The exogenous labelling generally consists in the cell’s incubation with the dye. The dye incorporation can occur with different uptake mechanisms, such as endocytosis, phagocytosis, active transporters, via adherence or diffusion. Askenasy and Farkas incubated bone marrow cells (BMCs) with PKH26 or PKH67 fluorescent membrane linkers. The exogenous labelling occurred with the encapsulation of the aliphatic fluorescent molecules into cell membrane lipid bilayer. With this approach, they were able to study the behaviour of hematopoietic cells transplanted into the recipient bone marrow stromal microenvironment by fluorescence microscopy (Askenasy and Farkas 2002)*.* Due to their large size, quantum dots or nanocrystal cannot pass the membrane of cells with the passive diffusion mechanism (Michalet et al. 2005). Therefore, the labelling of these particles with live cells can occur through several techniques such as electroporation, microinjection, transfection or endocytosis by the cells (Sun et al. 2014; Damalakiene et al. 2013)*.* In fluorescence the exogenous labelling of immune cells has been widely used for the rapid and efficient staining of cells. To overcome the autofluorescence generated from tissues are usually used fluorochromes with an emission in the near infrared (NIR) window (650–900 nm).

**Fig. 5:**
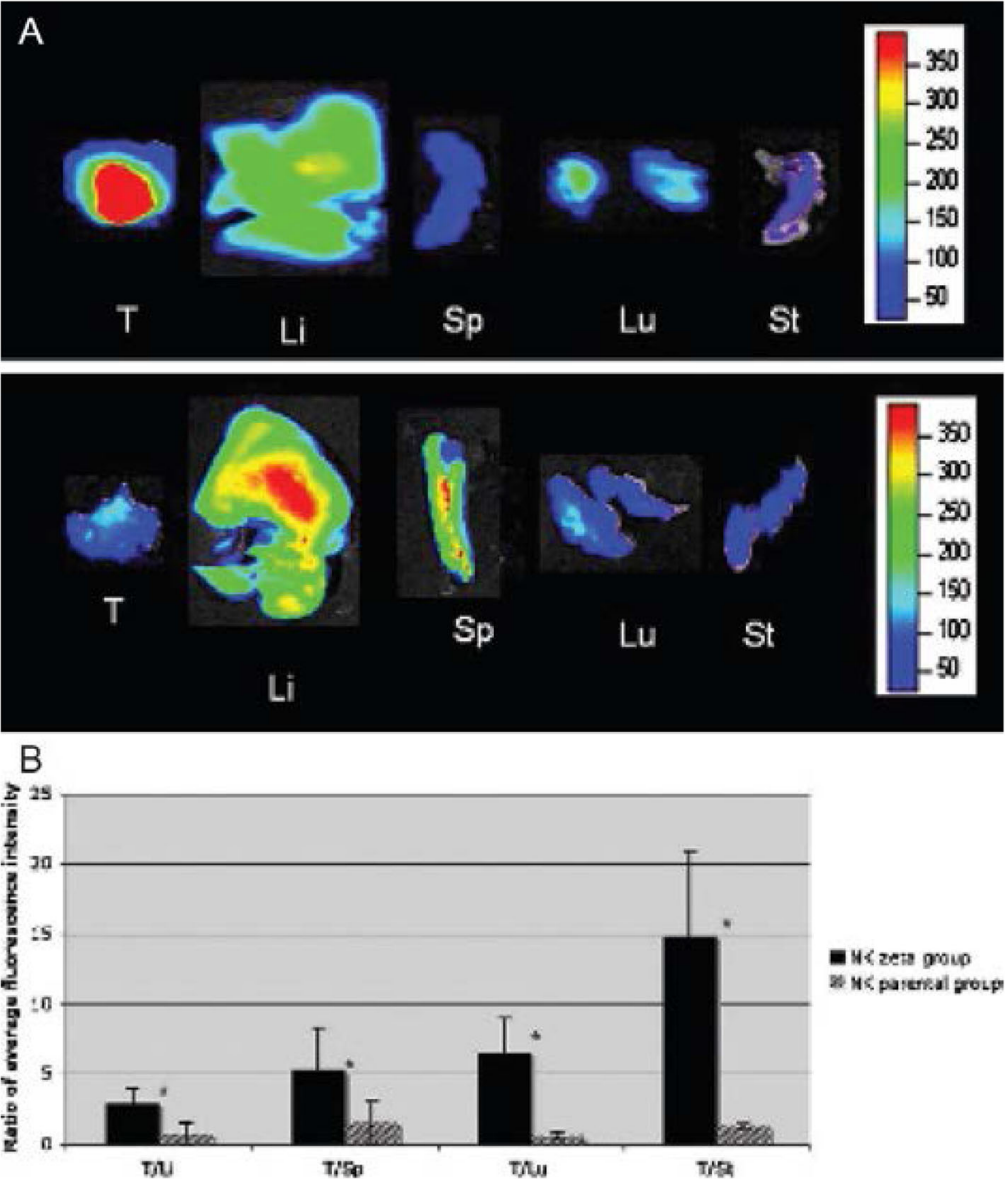
Ex vivo optical imaging (OI) study. OI of explanted tumors and organs of two representative animals at 24 h after NK92-scFv(MOC31)-zeta cell injection (upper panel) or NK-92 cell injection (lower panel). Following NK-92-scFv(MOC31)-zeta cell injection, a marked fluorescence of the tumor was noted, which was higher in intensity compared with the explanted organs. Following NK-92 cell injection, the tumor did not show an increased fluorescence. Li = liver; Lu = lungs; Sp = spleen; St = sternum; T = tumor. (adapted from Tavri et al.) (Tavri et al. 2009)

A near-infrared dye DiD (1,1-dioctadecyl-3,3,3,3-tetramethylindodicarbocyanine) was used to label NK-92 cells and NK-92-scFv(MOC31)-zeta cells functionalized to target the epithelial cell adhesion molecule (EpCAM) antigen on prostate cancer cells. As shown in Fig. 5, ex-vivo images show higher tumor infiltration of functionalized NK-92 cells. Fluorescence imaging was also compared with fluorescence microscopy to confirm the presence of functionalized NK-92 cells in tumor site compared to non-targeted cells (Tavri et al. 2009)*.*

The avidin-biotin system was used by Liu K. et al. to monitor the real time immunotherapy with CAR-T cells in glioma xenografts expressing EGFRvIII. They biotinylated the anti-EGFRvIII monoclonal antibody, 4G1 (biotin-4G1), and then bioengineered the expression of avidin-CAR on T cells. The optical imaging allowed them to assess both the ability of avidin-CAR-T cells to target tumor-expressing EGFRvIII cells and the correct time point for T cells adoptive transfer (Liu et al. 2015)*.*

The endogenous labelling consists in a genome manipulation inserting protein-encoding genes in the desired location to have a cell monitoring of endogenous activities (Fetter et al. 2015)*.* The transfection of DNA or RNA into animal cells can be obtained with transiently open pores of cells thorough electroporation or utilizing chemical substances (Li et al. 2013)*.*

The transduction of DNA or RNA is performed with viral vector carriers, as lentiviral, adenoviral and adeno-associated virus (AAV). Von der Haar et other researchers compared the transient transfection by electroporation with the lentiviral transduction for cell tracking of human mesenchymal stem cells derived from adipose tissue (AD-hMSCs). They also compared the use of a reporter gene such as enhanced green fluorescent protein (EGFP) with a synthetic fluorescent nonsense DNA. The results showed as the EGFP expression did not change the AD-hMSCs immunophenotype using the electroporation whereas this occurs with the lentiviral transduction. Also, the reporter gene expression resulted higher with the electroporation method than lentiviral. They concluded with the use of electroporation as the best method to introduce both nonsense DNA and EGFP gene reporter in AD-hMSCs (Von der Haar et al. 2019)*.*

Bioluminescence (BLI) exploits endogenous/indirect labelling with a gene transfer using the luciferase/luciferin biosystem (Jung et al. 2015)*.* The luciferin (substrate), administered intravenously, is oxidized by luciferase (enzyme) with emission of photons, without the need for light excitation.

BLI provides a higher sensitivity compared to fluorescent probes. The BLI signal has a high target-to-background ratio and also it is detected only from the site of luciferase expression, without the interferences from other cells or tissues. Then, the image can be acquired with a low wavelength and a maximum penetration depth of 3 cm.

Different luciferase proteins are available such as bacterial luciferase, Renilla luciferase (RLuc), firefly luciferase (FLuc) and Gaussia luciferase (GLuc), allowing simultaneous imaging of two different enzymes in the same animal. A real time imaging of stem cell differentiation with two different luciferases, RLuc and FLuc, were studied by Wang et al. to predict the response to a stem cell therapy. The encoding of two different reporter genes consented them to study both the proliferation and the survival of the injected mesenchymal stem cells, and their kinetics in endothelial differentiation (Wang et al. 2012). Edinger et al. used both the green fluorescent protein and BLI to study the entire progress of lymphoma disease and its response to ex vivo–expanded CD8+ natural killer (NK)–T cells. They revealed the timing of NK-T cells homing to tumor site, and its effectiveness as immune cell therapy (Edinger et al. 2003)*.* Tregs migration and proliferation were tracked using luciferase-expressing (luc+) mice model of bone marrow transplantation. The study showed as the induced inflammation by irradiation leads to migration and expansion of Tregs, providing a reduction in the proliferation of effector T-cells in the lymphoid organs. In addition, early infusion of Tregs, after a bone marrow transplant has been shown to protect the recipient from the GVHD (Nguyen et al. 2007). However, human applications of these approaches are limited by the need to modify the genetic code to make luciferase expressed by human cells. For this reason, BLI is limited to pre-clinical settings.

Moreovero, despite the usefulness of OI in pre-clinical studies, its approach in humans is limited also by the low penetration depth of photons.

## Future perspectives on labelling and tracking CAR cells in vivo

To improve and evaluating the efficacy of CAR-based immunotherapy, it is necessary to develop systems that allow better real-time monitoring of traffic, tissue distribution, survival and proliferation in immunosuppressive tumour microenvironments of these cells in vivo. Especially an imaging study of the tumor microenvironment will provide answers on the efficacy of these therapies. Using PET imaging to study the expression of immune checkpoints, such as anti-PD-1, already assessed by biopsies before immunotherapy, it will be possible to have an assessment of the immune status of the tumor and open the doors to new theragnostic approaches in the future. Currently studies are not underway with isotopes that allow overcoming the limits previously described for the direct labelling. In addition to the isotopes already mentioned, ^52^Mn chelated with oxine has been explored, but high cellular efflux of the isotope limits its use for prolonged cell tracking, as already discussed for zirconium-89 or copper-64 (Gawne et al. 2018). The few studies regarding cell labelling with ^64^Cu-gold nanoparticles through electroporation, but their use appears to be the most promising direct labelling technique. It is always difficult to make predictions, but when it will be possible to overcome the risks related to insertional oncogenes and regulatory problems related to these techniques the introduction of reporter genes in therapeutic cells will probably be the method with the highest sensitivity for long term monitoring of cell trafficking, proliferation and persistence. As previously described, this technique does not suffer from the loss of the labelling agent from the cells and the dilution effect and allows monitoring the cell proliferation rate as the signal increases with the number of new cells.

## Conclusions

In recent years new immune and cell-based therapies set new standards in terms of diagnostic accuracy to properly predict and evaluate therapy efficacy. The major challenge in patients eligible for these therapies is to distinguish between true- and pseudo- progression and this can be achieved only by in vivo imaging of effector cells. Imaging of different immune subpopulation would be of great importance not only for therapy decision-making, but also to assess therapy efficacy, thus increasing success rates of immunotherapies.

MRI, optical and nuclear medicine imaging are three modalities with different pros and cons (Table 1). Whereas optical imaging offers great potential in a pre-clinical setting, MRI and nuclear medicine imaging are more suitable for human studies. Despite a high spatial resolution, reported sensitivity and specificity for MRI are generally lower if compared to nuclear medicine imaging. Furthermore, the iron content in healthy or pathologic tissues influences MR image interpretation. On the contrary, nuclear medicine imaging has high sensitivity and specificity, but low spatial resolution compensated by hybrid imaging in combination with CT.

**Table 1:** Imaging modalities for in vivo tracking of immune cells

Heading	Modality	Advantages	Disadvantages
**Nuclear medicine imaging**	PET	High sensitivity and specificity; no depth limit; clinically applicable; quantitative	Ionizing radiation exposure; expensive; relatively low spatial resolution 5 mm; no standardized cell labeling method
	SPECT	High sensitivity and specificity; no depth limit; clinically applicable; cell tracking at late time points	Ionizing radiation exposure; expensive; long scan times; relatively low spatial resolution 10 mm; no standardized specific cell labeling method
**Magnetic Resonance imaging**	MRI	High resolution (more than 0.1 mm); no ionizing radiation exposure; clinically applicable; possible quantification (indirect)	Lower sensitivity than PET/SPECT; high costs; contrast agents interference with cells; long scan times
**Optical imaging**	MI	Visualization of cell processes and interactions; time-lapse imaging	Photobleaching, phototoxicity; diffraction limit of light; no translational potential for in-vivo imaging
	BLI	Short acquisition time; high sensitivity (100 cells); high signal-to-noise ratio (no background signal)	No translational potential for in-vivo imaging; diffraction and absorption of light by tissues; immunogenicity or gene silencing; erroneous readouts; signal quantification; half-life and stability of enzymes; limited penetration depth (3 cm)
	FLI	Low cost; radiation free; easy labeling method; real-time	Tissue autofluorescence and absorption; limited penetration depth (1 cm); poor spatial resolution (photon scattering); limited quantification

PET/MRI hybrid imaging could certainly overcome limitation of both modalities and offer a powerful tool with great spatial resolution and diagnostic accuracy (Signore et al. 2017; Glaudemans et al. 2012).

Regardless of the imaging method, direct cell labelling is a simple approach, but still require ex vivo manipulation of cells and it is limited by radiotoxicity and efflux of the labelling agent from the cells that cause altered biodistribution and image misinterpretation. Ex vivo labelling approaches require a relatively high volume of blood from patients and sometimes this could be difficult to obtain in clinical setting from patients with severe pathologies. The same limitation applies pre-clinical studies with small animals such as murine models.

Furthermore, due to the possible alteration of the cells subject to ex vivo labelling, it is crucial to ensure the viability of these cells, and any change in cell surface markers that could affect their functionality. Indirect cell labelling with reporter genes requires ex vivo and genetic manipulation of cells with the need of well-trained personnel and special facilities and equipment. This approach offers a high T/B ratio and the possibility to track specific immune subsets, but safety and regulatory issues may limit its application in humans. In vivo indirect cell labelling using radiolabelled mAbs, peptides or proteins seems the best strategy, so far, with the highest translational potential. However, it may suffer of high background activity and the difficulty to select a specific cell subtype to target, unless a highly specific antigen is selected.

## Data Availability

Not applicable.

## Abbreviations

AAV: Adeno-associated virus
AICD: Activation-induced cell death
APCs: Antigen-presenting B cells
ATMP: Advanced therapy medicinal product
BLI: Bioluminescence
BMCs: Bone marrow cells
CAR: Chimeric antigen receptor
CT: Computed tomography
DiD: 1,1-dioctadecyl-3,3,3,3-tetramethylindodicarbocyanine
EBV: Epstein-Barr virus
EGFP: Enhanced green fluorescent protein
FDA: Food and drug administration
FDG: Fluorodeoxyglucose
IgSF: Immunoglobulin superfamily
[^18^F]FEAU: 2-deoxy-2-[^18^F]5-ethyl-1-D-arabinofuranosyluracil
[^18^F]FHBG: 9-[4-[^18^F]3-(hydroxymethyl)-butyl]guanine
[^18^F]FIAU: 2-deoxy-2-[^18^F]5-iodo-1-D-arabino-furanosyluracil
FLuc: Firefly luciferase
GLuc: Gaussia luciferase
GVHD: Graft versus host disease
HMPAO: Exametazime
ITAMs: Immunoreceptor tyrosine-based activation motifs
ICOS: Inducible T-cell COStimulator
mAb: Monoclonal antibody
MRI: Magnetic resonance imaging
NIS: Sodium-iodine symporter
NK: Natural killer
PBMCs: Peripheral blood mononuclear cells
PET: Positron emission tomography
PTSM: Pyruvaldehyde-bis(N4-methylthiosemicarbazone)
RLuc: Renilla luciferase
SPECT: Single photon emission tomography
SPIO: Superparamagnetic iron oxide
^99m^Tc-IL2: ^99m^Tc-interleukin-2
TNFRSF: Tumor-necrosis factor receptor super family
TRUCKs: Universal cytokine killing T cells
WBC: White blood cells
